# The International Medical Annual

**Published:** 1890-05

**Authors:** 


**The International Medical Annual** and Practitioner's Index for 1890. Edited by P. W. WILLIAMS, M. D., Secretary of Staff, assisted by a corps of thirty-six collaborators—European and American—specialists in their several departments. 600 octavo pages. Illustrated. $2.75. E. B. Treat, publisher, 5 Cooper Union, New York.

The eighth yearly issue of this handy reference manual is at hand and is up to the standard of former editions. It gives in a brief but careful résumé of the important additions to medical literature during the past year, what cannot but be of great importance to the physician striving to be abreast of the times. Under miscellaneous are included two very important articles on Sanitary Science and Life Assurance, besides a list of the principal medical works published during the past year, chiefly American, and a list of the private asylums and homes for the insane, feeble-minded, inebriates, etc. The typographical work is neatly done.

